# Supplementary material for: Sexually dimorphic role for insular perineuronal nets in aversion-resistant alcohol consumption
Source: Front Psychiatry. 2023 Feb 28;14:1122423. doi: 10.3389/fpsyt.2023.1122423 (PMC10011443; doi:10.3389/fpsyt.2023.1122423)
Supplement: Supplementary file 1 [file Data_Sheet_1.PDF]

Supplementary Material

Sexually Dimorphic Role for Insular Perineuronal Nets in Aversion-Resistant Alcohol Consumption

Luana Martins de Carvalho[1#], Hu Chen[1], Mason Sutter[1] and Amy W. Lasek[1*#]

[1]Center for Alcohol Research in Epigenetics and Department of Psychiatry, University of Illinois at Chicago, Chicago, IL United States

[#]Current affiliation: Department of Pharmacology and Toxicology, Virginia Commonwealth University, Richmond, VA United States

*** Correspondence:** Amy.Lasek@vcuhealth.org

1. Supplementary Materials and Methods

1.1 Ethanol drinking procedure and sample collection for western blot

A one-bottle DID experiment was done with 20% ethanol or water (control) for 4 h in one day to test the effect of acute ethanol exposure on aggrecan and brevican protein levels in the insula by western blot. Male mice consumed 3.4 ± 0.29 g/kg and females consumed 6.4 ± 0.95 g/kg ethanol during the 4 h session. Insula samples were collected immediately after the drinking session. The tissue punch size was 1 mm and included all layers of the anterior insula, centered at ~1.5 mm anterior to bregma. White matter was not included in the punch.

1.2 Western blot procedure

Frozen tissue punches containing the insula were manually homogenized in ice cold 1X RIPA buffer (Cell Signaling Technology, Danvers, MA) containing 1X Halt protease inhibitor cocktail (Thermo Fisher Scientific). Protein concentrations were determined using the Pierce BCA Protein Assay Kit (Thermo Fisher Scientific). Equal amounts of protein (15 µg) were separated by SDS-PAGE on precast Novex 4%–12% Tris-glycine gels (Thermo Fisher Scientific) and transferred to nitrocellulose membranes. Membranes were blocked with bovine serum albumin in Tris-buffered saline (20 mM Tris, 150 mM NaCl, pH 7.4). Membranes were then incubated with anti-aggrecan (#AB1031, Millipore Sigma, 1:1000) or anti-brevican (#610894, BD Biosciences, 1:2000) and anti-β-actin (#A5441, Millipore Sigma, 1:16,000) antibodies. Secondary antibodies were donkey anti-mouse IgG DyLight 680 (#SA5-10170, Thermo Fisher Scientific, 1:20,000) and donkey anti-rabbit IgG DyLight

800 (#SA5-10044, Thermo Fisher Scientific 1:5000). Blots were imaged on an Odyssey Fc Dual-Mode Imaging system (LI-COR) and analyzed using Image Studio Lite (LI-COR).

2. Supplementary Results

To confirm the sex difference in PNNs and to determine if acute ethanol exposure affects the expression of PNN proteins, we performed western blots on insula homogenates from male and female mice using antibodies to aggrecan and brevican, proteoglycan components of PNNs. Mice underwent a one-bottle DID procedure for one 4 h session with ethanol or water as a control prior to dissecting the insula. Aggrecan and brevican protein levels were significantly higher in the insula of females compared to males independently of whether mice drank ethanol or water (Supplementary Figure 1, aggrecan: sex, $F_{(1, 20)} = 7.03$, $P = 0.015$; Fig. 2F, brevican: sex, $F_{(1, 20)} = 9.84$, $P = 0.0052$). A single ethanol drinking session did not alter aggrecan or brevican protein levels.

3. Supplementary Figure 1

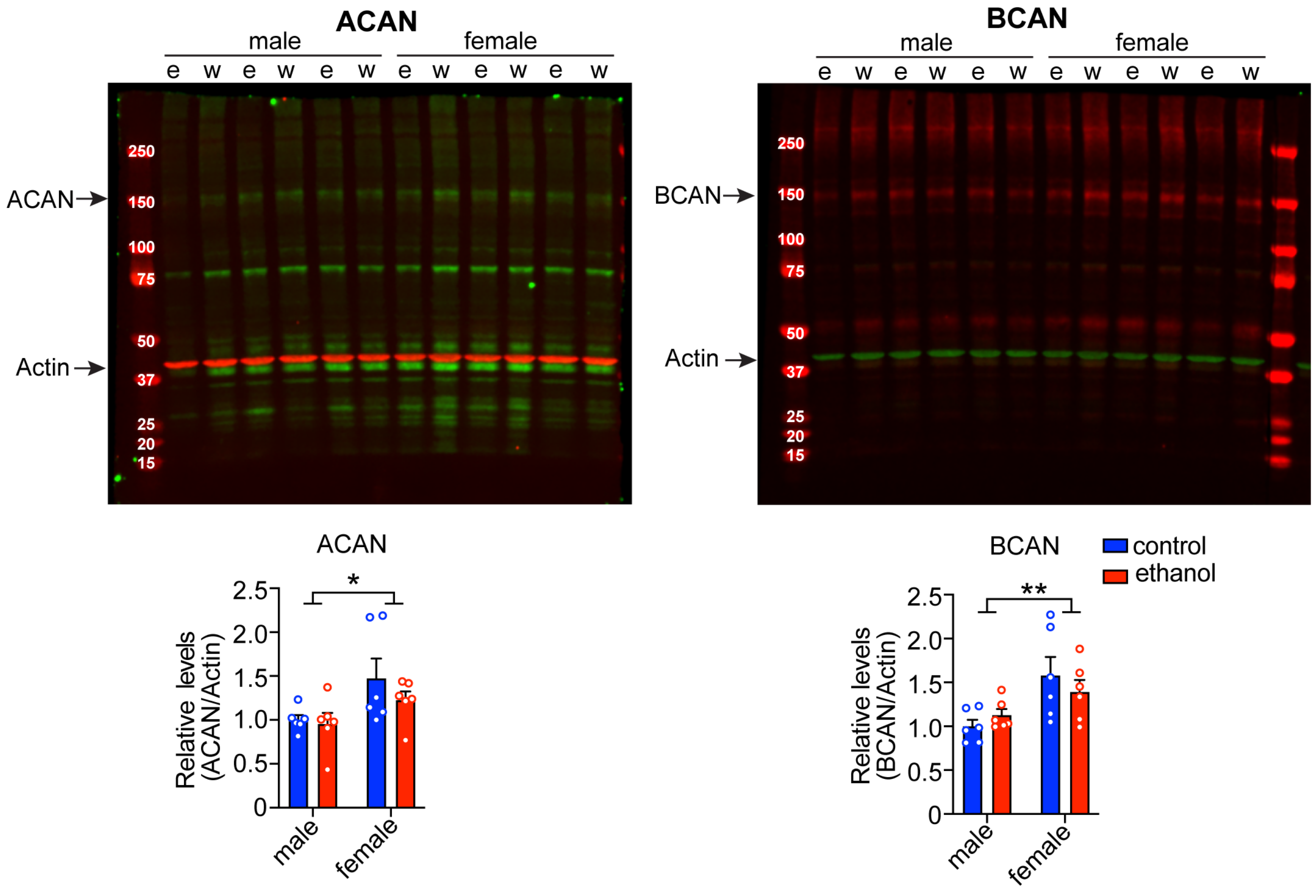

Supplementary Figure 1. Representative aggrecan and brevican western blot images. Aggrecan (ACAN) is on the left and brevican (BCAN) is on the right. Molecular weight markers were loaded in the in the first well of the gel and the molecular weight (in kDa) is indicated in white text over

each band. Arrows point to ACAN, BCAN, and actin bands that were quantified. e, ethanol treated; w, water treated. Note that the ACAN band that was quantified represents a cleaved ~150 kDa form and the BCAN band that was quantified represents the full-length non-glycosylated form. Graphs below each gel show the quantified results, n=6 per sex per treatment. ACAN and BCAN protein levels are relative to β-actin band intensity as a loading control. *$p<0.05$ and **$p<0.01$, main effect of sex by two-way ANOVA.
